# High-throughput, quantitative assessment of the effects of low-dose silica nanoparticles on lung cells: grasping complex toxicity with a great depth of field

**DOI:** 10.1186/s12864-015-1521-5

**Published:** 2015-04-18

**Authors:** Cédric Pisani, Jean-Charles Gaillard, Virginie Nouvel, Michaël Odorico, Jean Armengaud, Odette Prat

**Affiliations:** CEA, IBEB, SBTN, F-30207 Bagnols-sur-Cèze, France; International Consortium for the Environmental Implications of Nanotechnology (iCEINT), Aix-en-Provence, France

**Keywords:** Transcriptomics, Proteomics, Toxicogenomics, Nanoparticles, Silica, Cytotoxicity, Adverse outcome pathways, Cellular impedance

## Abstract

**Background:**

The toxicity of manufactured fumed silica nanoparticles (NPs) remains poorly investigated compared to that of crystalline silica NPs, which have been associated with lung diseases after inhalation. Amorphous silica NPs are a raw material for manufactured nanocomposites, such as cosmetics, foods, and drugs, raising concerns about their potential toxicity.

**Results:**

The size of the NPs was determined by dynamic light scattering and their shape was visualized by atomic force microscopy (10 ± 4 nm). The pertinent toxicological concentration and dynamic ranges were determined using viability tests and cellular impedance. We combined transcriptomics and proteomics to assess the cellular and molecular effects of fumed silica in A549 human alveolar epithelial cells. The “no observed transcriptomic adverse effect level” (NOTEL) was set to 1.0 μg/cm^2^, and the “lowest observed adverse transcriptional effect level” (LOTEL) was set at 1.5 μg/cm^2^. We carried out genome-wide expression profiles with microarrays and identified, by shotgun proteomics, the exoproteome changes in lung cells after exposure to NP doses (0.1, 1.0, 1.5, 3.0, and 6.0 μg/cm^2^) at two time points (24 h and 72 h). The data revealed a hierarchical, dose-dependent cellular response to silica NPs. At 1.5 μg/cm^2^, the Rho signaling cascade, actin cytoskeleton remodeling, and clathrin-mediated endocytosis were induced. At 3.0 μg/cm^2^, many inflammatory mediators were upregulated and the coagulation system pathway was triggered. Lastly, at 6.0 μg/cm^2^, oxidative stress was initiated. The proteins identified in the extracellular compartment were consistent with these findings.

**Conclusions:**

The alliance of two high-throughput technologies allowed the quantitative assessment of the cellular effects and molecular consequences of exposure of lung cells to low doses of NPs. These results were obtained using a pathway-driven analysis instead of isolated genes. As in photography, toxicogenomics allows, at the same time, the visualization of a wide spectrum of biological responses and a “zoom in” to the details with a great depth of field. This study illustrates how such an approach based on human cell culture models is a valuable predictive screening tool to evaluate the toxicity of many potentially harmful emerging substances, alone or in mixtures, in the framework of future regulatory reinforcements.

**Electronic supplementary material:**

The online version of this article (doi:10.1186/s12864-015-1521-5) contains supplementary material, which is available to authorized users.

## Background

With the growing commercialization of nanotechnology products, human exposure to manufactured nanoparticles (NPs) is increasing, raising concerns about their potential toxicity. Synthetic, amorphous silica NPs are a raw material for the manufacture of many nanocomposites, such as cosmetics, foods, drugs, and printing ink additives, on a large industrial scale [1]. The toxicity of crystalline silica, the inhalation of which is involved in many lung diseases, such as silicosis, lung cancer, chronic obstructive pulmonary diseases, and pulmonary tuberculosis, has been widely studied [1,2]. However, few studies have been performed on amorphous silica, because it exists in many diverse forms of natural or synthetic origin [3,4]. Industrial processes use synthetic amorphous silica nanoparticles for the easy control of size, shape and physical properties. Fumed (or pyrogenic) silica is prepared by the oxidation of silicon tetrachloride at elevated temperature, leading to a very low bulk density and high specific surface area nanopowder (200 m^2^/g for AEROSIL® 200 NPs). Fumed silica is used extensively as strengthening filler and thickening agent in a wide variety of products, such as foodstuffs, paints, printing inks (toners) and adhesives. It also serves as a desiccant and is frequently added to cosmetics and toothpastes due to its respective light-diffusing and mild-abrasive properties. Along with applications in polyester, silicone, paints, and coatings, hydrophilic fumed silica AEROSIL® products are used with increasing success in high-technology fields such as electronics and optical fiber industries. Fumed silica poses an inhalation risk due to its extremely fine structure and the workers synthesizing or using it may be the first population exposed.

Some *in vivo* toxicological studies have been carried out with AEROSIL products. Reuzel *et al.* showed that AEROSIL® 200 (30 mg/m^3^) led to a clear dose–response relationship and to the accumulation of alveolar macrophages [5]. Of the amorphous silicas examined, AEROSIL® 200 induced the most severe changes in the lungs of rats, which only partly recovered. Previous *in vitro* studies also showed that amorphous silica nanoparticles were toxic to lung cells [4,6,7].

While *in vivo* toxicity assays in animals remain the gold-standard methodology for evaluating the toxicity of a compound, the existence of a growing number of manufactured products mandates an urgent requirement for alternative assays based on high-throughput, cell-based methodologies. Toxicogenomics, including in its broader scope transcriptomics and proteomics, is a promising tool not only for monitoring the toxicity of substances using human cell line assays but also for investigating and documenting the modes of action of unknown chemicals [8-12]. In this domain, Lobenhofer [13] developed a new concept that could potentially be a new, sensitive, and informative parameter to be systematically documented for risk assessment and comparison [14]. This parameter is the highest concentration of a given chemical for which no effect on the transcriptome is observed and is named the “no observed transcriptional effect level” (NOTEL). Proteomics has, in addition, been proposed as a complementary approach to study the cellular response to toxicants [15]. Specifically, the analysis of human cell line secretomes appears to be a promising source of biomarkers documenting their physiological state in response to chemicals [16].

Here, we have focused on the cellular effects induced by fumed silica NPs (AEROSIL® 200 NPs) in A549 lung epithelial cells. For this, we established the transcriptomic dose–response curve over a range of silica concentrations and accurately determined the NOTEL threshold using commercial human 4x44k Gene Expression Microarrays. We also performed, by high-resolution shotgun proteomics, an analysis of the secretome of A549 cells after exposure. This new strategy allowed the highlighting, in a dose-dependent manner, of the significant cellular and molecular modifications triggered by the exposure to fumed silica NPs.

## Results

### Characterization of silica NPs

**Figure 1:**
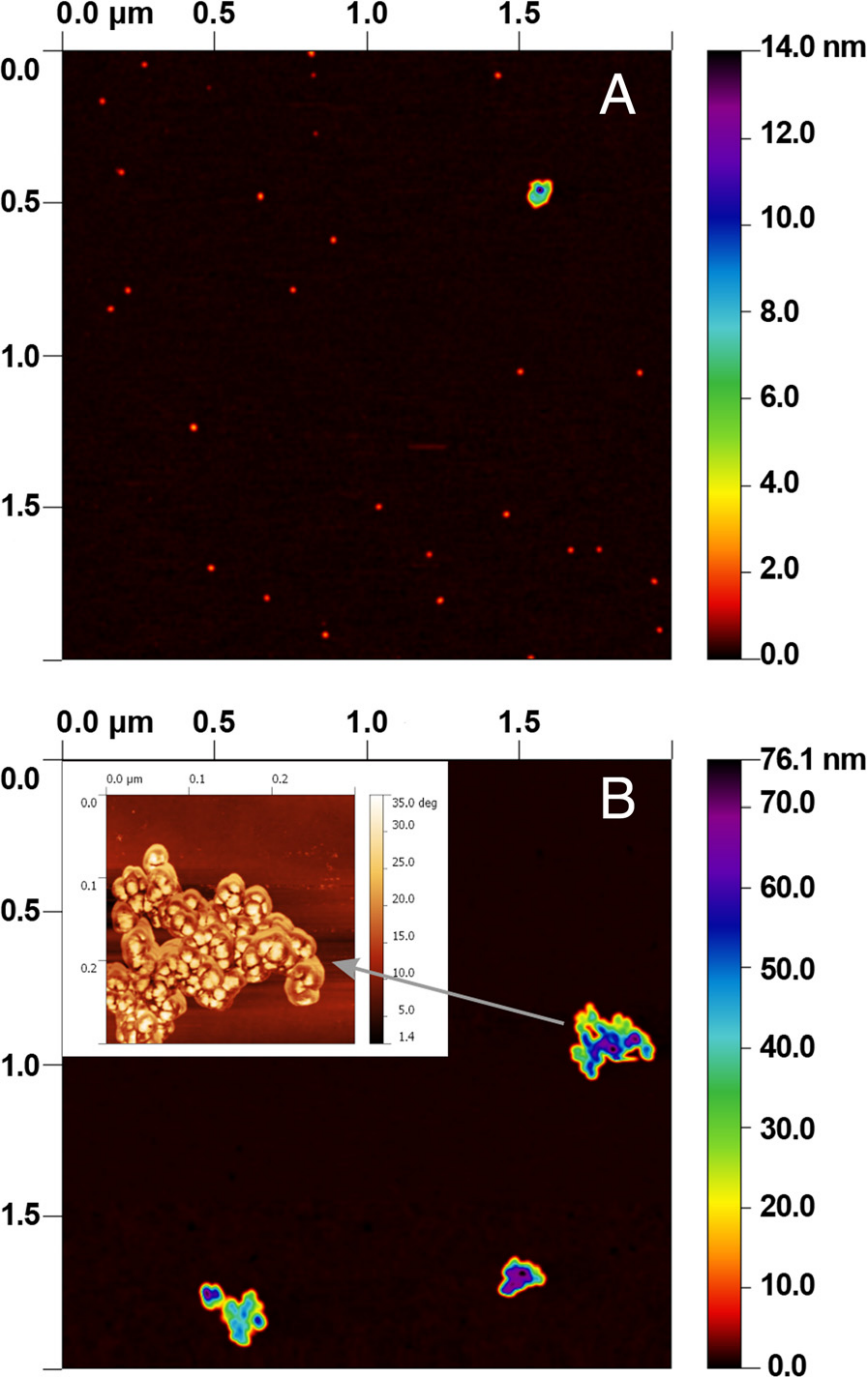
AFM characterization of fumed silica AEROSIL® 200 NPs. 5 μL of AEROSIL® 200 NPs suspension (10 μg/mL in water) were deposited on freshly cleaved mica then dried under vacuum. AFM imaging was recorded with a Multimode and Nanoscope V instrument (Veeco, Santa Barbara, CA, USA). The scale of false colors spectrum code ranges from 0 to maximum height (in nm) using Gwyddion software. **A)** AFM imaging shows average size of individual disperse disks with diameter 10 ± 5 nm and thickness 2 nm ± 0.3 nm. An agglomerate is observed in the upper right side. **B)** Details of AFM imaging of SiO_2_ NPs agglomerate in the same sample. The average size of agglomerates is 160 ± 90 nm. In the inset, a zoom of a SiO_2_ agglomerate, recorded with peak force phase, reveals details of individual members clustered in the agglomerate.

The silica NPs were first characterized in terms of aggregation and size. In DMEM/F12 culture medium comprising 10% fetal bovine serum (FBS), the average hydrodynamic diameter of the NPs was 10 ± 4 nm, as measured by dynamic light scattering (DLS). This value is close to the value provided by the manufacturer (12 nm). NPs in serum-free medium were in an agglomerated form with a mean size, evaluated by DLS, close to 350 nm (±8 nm). Atomic Force Microscopy (AFM) showed disperse disks with diameter 10 ± 5 nm and thickness 2 ± 0.3 nm (Figure 1A) and confirmed the existence of size heterogeneity. Figure 1B shows a typical 160 ± 90 nm agglomerate, where individual base units are clearly visible.

### Viability tests and real-time cellular impedance

**Figure 2:**
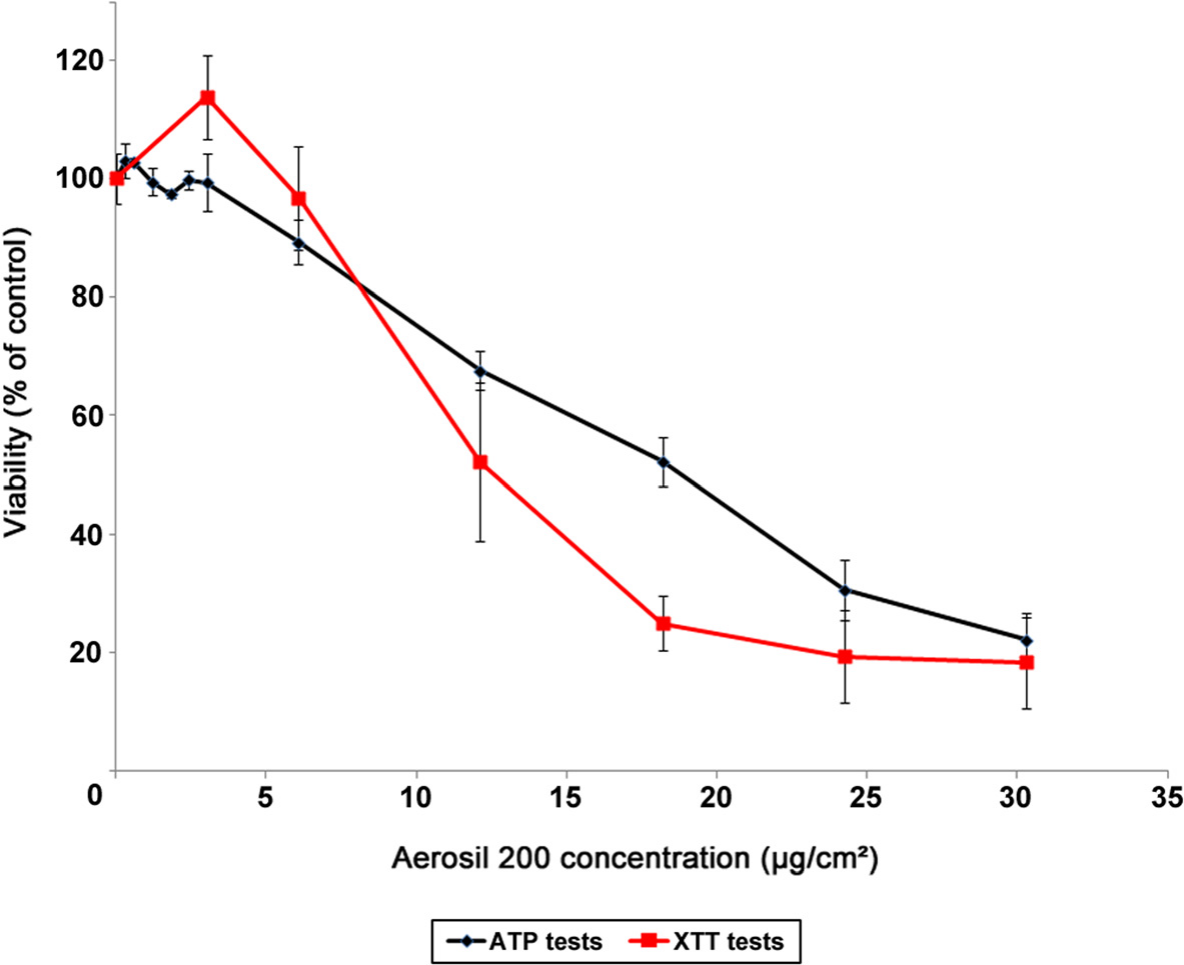
Cell viability of A549 cells exposed to fumed silica AEROSIL® 200 NPs. After 24 h exposure to silica AEROSIL® 200 NPs, the viability of A549 cells was determined using the ATP test (black line) (Cell Titer Glo, Promega) and the XTT test (red line) (TOX2, Sigma). The results are expressed as the mean percent viable cells ± SD (n = 3) compared with unexposed cells.

We evaluated the cytotoxicity of SiO_2_ NPs in A549 human alveolar epithelial cells in serum-free medium. Two different toxicity assays, ATP (Cell Titer Glo, Promega) and XTT (TOX-2, Sigma), were carried out. These two assays comprised two different reporting systems – fluorescence and optical density, respectively – and were performed in parallel to identify potential bias due to possible interference from the nanoparticles. The ATP test based on the intracellular ATP content measurement is considered as a very sensitive viability test. The XTT test is based on the activity of succinate dehydrogenase, a mitochondrial enzyme. This test was chosen to avoid the more standard MTT test. Indeed, the formation of insoluble MTT formazan in this assay is often interlaced with NP fibers or agglomerates, thus inhibiting its solubilization by the solvent [17,18]. As shown in Figure 2, both the ATP and XTT tests produced similar dose–response curves over the concentration range tested, from 0.3 - 30.3 μg/cm^2^ of cell surface. AEROSIL® 200 NPs were cytotoxic from 3.0 - 30.3 μg/cm^2^. The no observed adverse effect level (NOAEL) for these NPs was estimated at 1.5 μg/cm^2^ and the lowest value for which an effect on cell viability was observed (LOAEL) was established at 3.0 μg/cm^2^ silica NPs (Figure 2).

**Figure 3:**
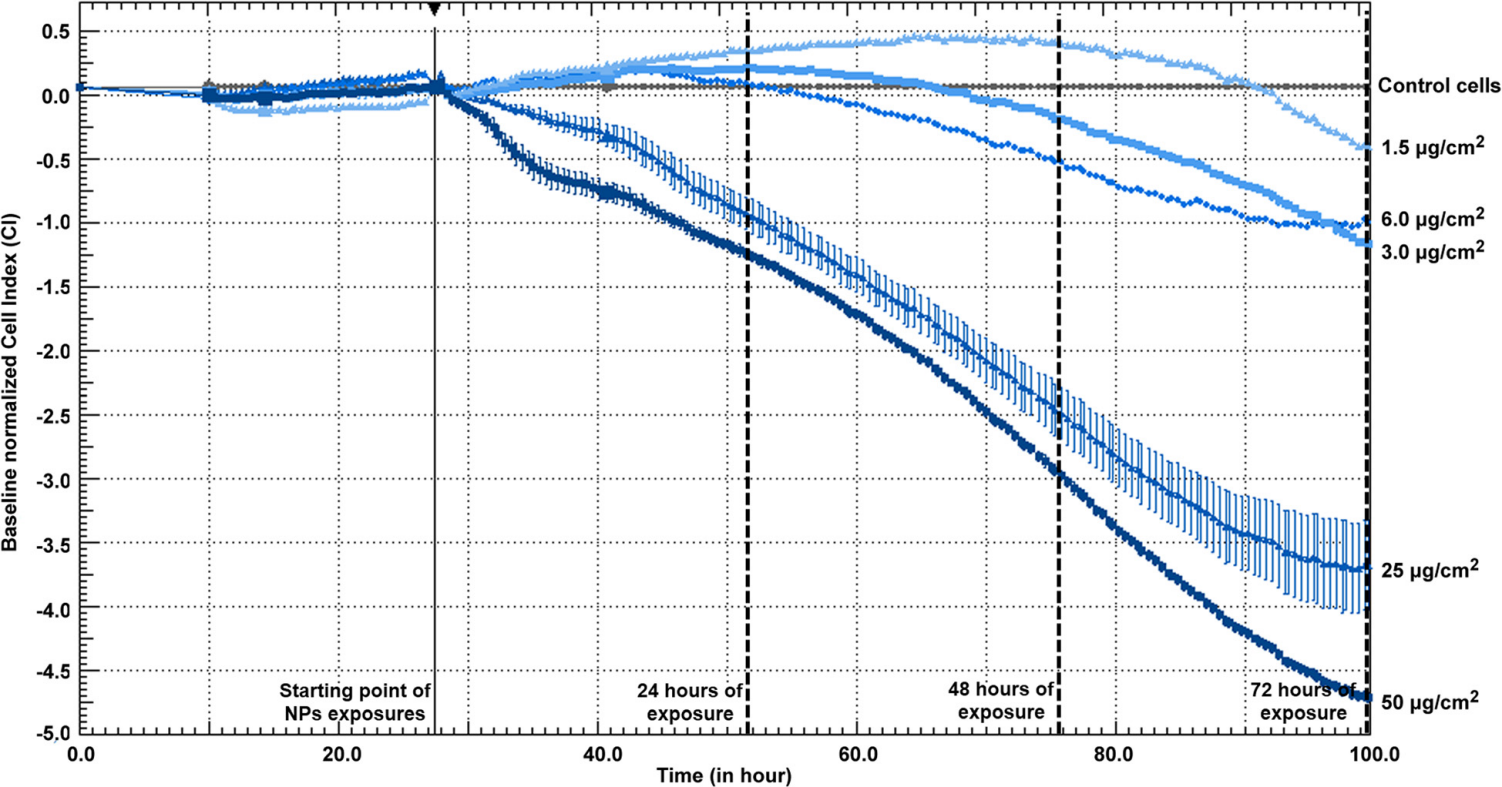
XCELLigence experiment. Real-time cell index (CI) monitoring of A549 cells (n = 3) exposed to 1.5, 3.0, 6.0, 25, and 50 μg/cm^2^ fumed silica AEROSIL® 200 NPs for 72 h. The black arrow represents the starting point of exposure. Cell index was normalized on baseline (control cells) to ensure inter-dose comparison. The highest concentrations (i.e. 25 and 50 μg/cm^2^) induced a drastic CI decrease compared to control cells. The lowest concentrations (1.5, 3.0 and 6.0 μg/cm^2^) indicated a biphasic CI evolution with an early increase followed by a decrease up to the end of the experiment.

An xCELLigence RTCA instrument was used to carry out real-time cell monitoring after exposure to NPs (Figure 3). The highest concentrations (i.e. 25 and 50 μg/cm^2^) induced a drastic decrease in cell index (CI) compared with control cells. The lowest concentrations (1.5, 3.0, and 6.0 μg/cm^2^) indicated a biphasic CI evolution with an early increase, followed by a decrease up to the end of the experiment.

### Omics-based probing of the silica NP cellular effects

#### Correlation between gene expression profiles and viability assays

**Figure 4:**
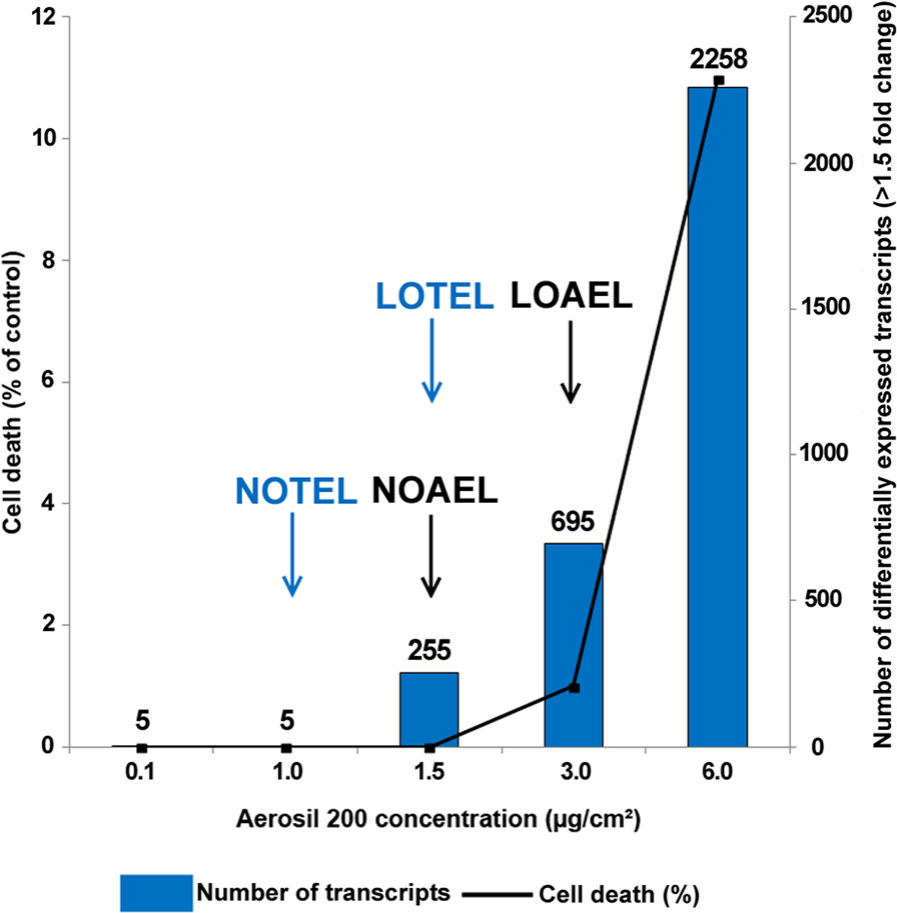
Transcriptomic dose–response curve compared with ATP assay. A549 cells (n = 3) were exposed for 24 h to increasing concentrations of fumed silica AEROSIL® 200 NPs (0.1, 1.0, 1.5, 3.0, and 6.0 μg/cm^2^). Blue histograms represent the number of differentially expressed genes after statistical analysis with Genespring software (Agilent). The black curve indicates the cell death percentage obtained with the ATP viability test related to the concentration of silica nanoparticles. The NOTEL (no observed transcriptional effect level) was determined to be 1.0 μg/cm^2^ and the LOTEL (lowest observed transcriptional effect level) as 1.5 μg/cm^2^. The NOAEL (no observed adverse effect level) was determined as 1.5 μg/cm^2^ and the LOAEL (lowest observed adverse effect level) at 3.0 μg/cm^2^.

**Figure 5:**
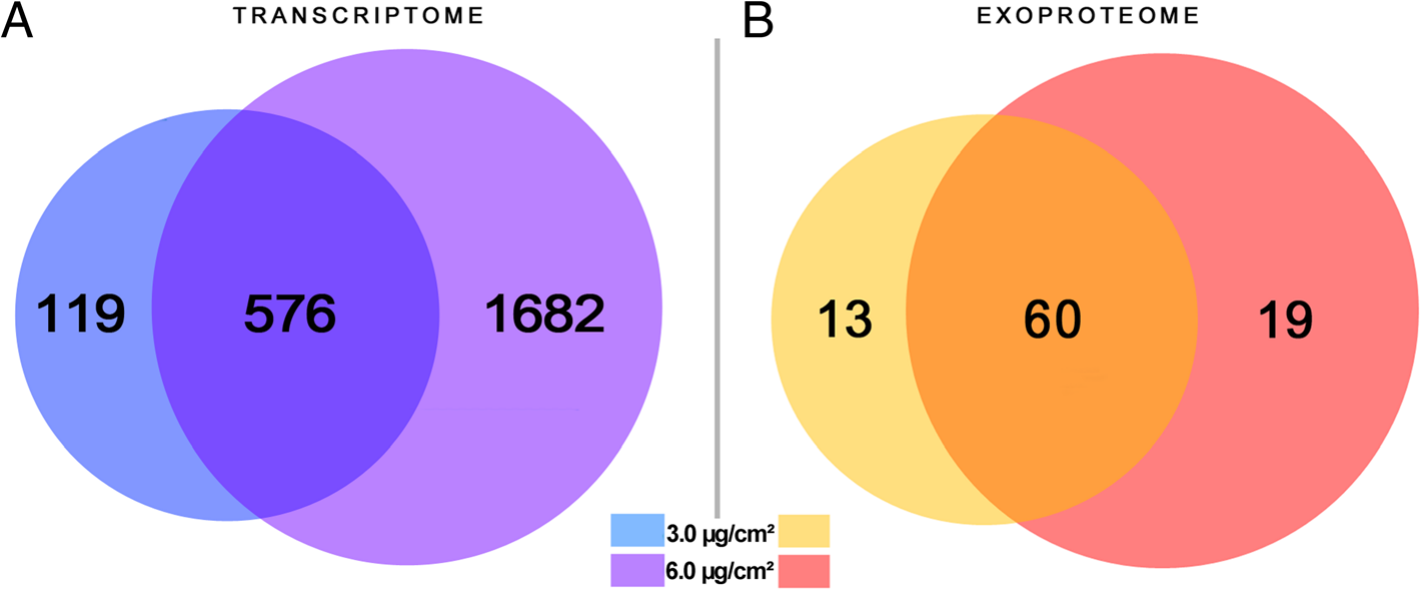
Venn diagrams of significantly modulated genes and proteins (p-value ≤ 0.05). A549 cells (n = 3) were exposed for 24 h to increasing concentrations of fumed silica AEROSIL® 200 NPs (3.0 and 6.0 μg/cm^2^). **A)** Number of modulated genes. **B)** Number of modulated proteins in supernatants. More than 80% of the modulated molecules present at 3.0 μg/cm^2^ were also modulated at 6.0 μg/cm^2^ in both omics analyses.

Gene expression changes depending on silica NP concentrations were evaluated in three biological replicates with Agilent Human V2 4x44K Microarrays. Figure 4 shows the number of differentially expressed genes detected in response to 0.1, 1.0, 1.5, 3.0, and 6.0 μg/cm^2^ AEROSIL® 200 silica NPs during 24 h exposure. A dose-dependent response to silica NPs was observed, ranging from 5 to 2,258 significantly differentially modulated transcripts compared with controls, with a fold change of at least 1.5 and p-value < 0.05. The lists of modulated transcripts including fold changes and p-values are presented in Additional file 1: Table S1. These results showed a strong correlation between the numbers of modulated genes and the cell death percentage established with viability tests (ATP, XTT). From these results, the NOTEL was set at 1.0 μg/cm^2^, with two successive concentrations (0.1 and 1.0 μg/cm^2^) giving no significantly differentially expressed genes as the criterion (Figure 4). The LOTEL was set at 1.5 μg/cm^2^, with 255 transcripts as compared with controls. Figure 5A shows the numbers of differentially expressed transcripts obtained with 3.0 and 6.0 μg/cm^2^ silica NPs, and their overlaps. A strong concordance of the results is represented by the Venn diagram (Figure 5A). At 24 h, more than 80% of the transcripts modulated at 3.0 μg/cm^2^ were also modulated at 6.0 μg/cm^2^. Finally, the 3.0 and 6.0 μg/cm^2^ doses were also analyzed with whole genome microarrays after 72 h exposure, to further identify potentially conserved adverse outcome pathways. We found 983 and 3,221 modulated transcripts at 3.0 and 6.0 μg/cm^2^, respectively. After 72 h exposure, 88% of the transcripts modulated at 3.0 μg/cm^2^ were also modulated at 6.0 μg/cm^2^.

#### Exoproteome changes upon silica NP addition

To determine which secreted proteins were modulated upon silica NP exposure to 3.0 and 6.0 μg/cm^2^ doses at 24 h, we collected the supernatants of cell cultures from the transcriptomic experiments. This ensured that the data from both of these omics-based approaches were fully comparable. After protein extraction and trypsin proteolysis of the three biological replicates at the three conditions (zero, 3.0, and 6.0 μg/cm^2^ NPs), the resulting peptides were analyzed by shotgun proteomics, i.e. nano-LC resolution on a reverse phase coupled to tandem mass spectrometry operated with an Orbitrap mass analyzer. A total of 8,276 MS/MS spectra were assigned with confidence to human proteins (p-value ≤ 0.05) taking the nine samples into consideration, indicating the presence of 145 proteins (Additional file 2: Table S2). Figure 5B reports the number of significantly modulated proteins detected with this approach in the extracellular medium upon exposure to silica NPs. A total of 72 and 79 proteins were significantly modulated with 3.0 and 6.0 μg/cm^2^ at 24 h, respectively, with a fold change of at least 2.0 (p-value ≤ 0.05). As for transcriptomics, the Venn diagram showed a good correlation of the results between silica NP doses, with more than 80% of the proteins that were modulated at 3.0 μg/cm^2^ also modulated at 6.0 μg/cm^2^ (Figure 5B).

### Canonical pathway analysis

#### Transcriptomic analysis

We identified the most relevant canonical pathways disturbed after 24 and 72 h exposure at all NP doses using the Ingenuity Pathway Analysis tool. Each canonical pathway is constituted of a finite number of genes. For each dose and time point, we calculated a ratio indicating the percentage of altered genes in our dataset belonging to a given canonical pathway. These pathways are all significant according to a Fisher statistical test (p-value < 0.05).

**Figure 6:**
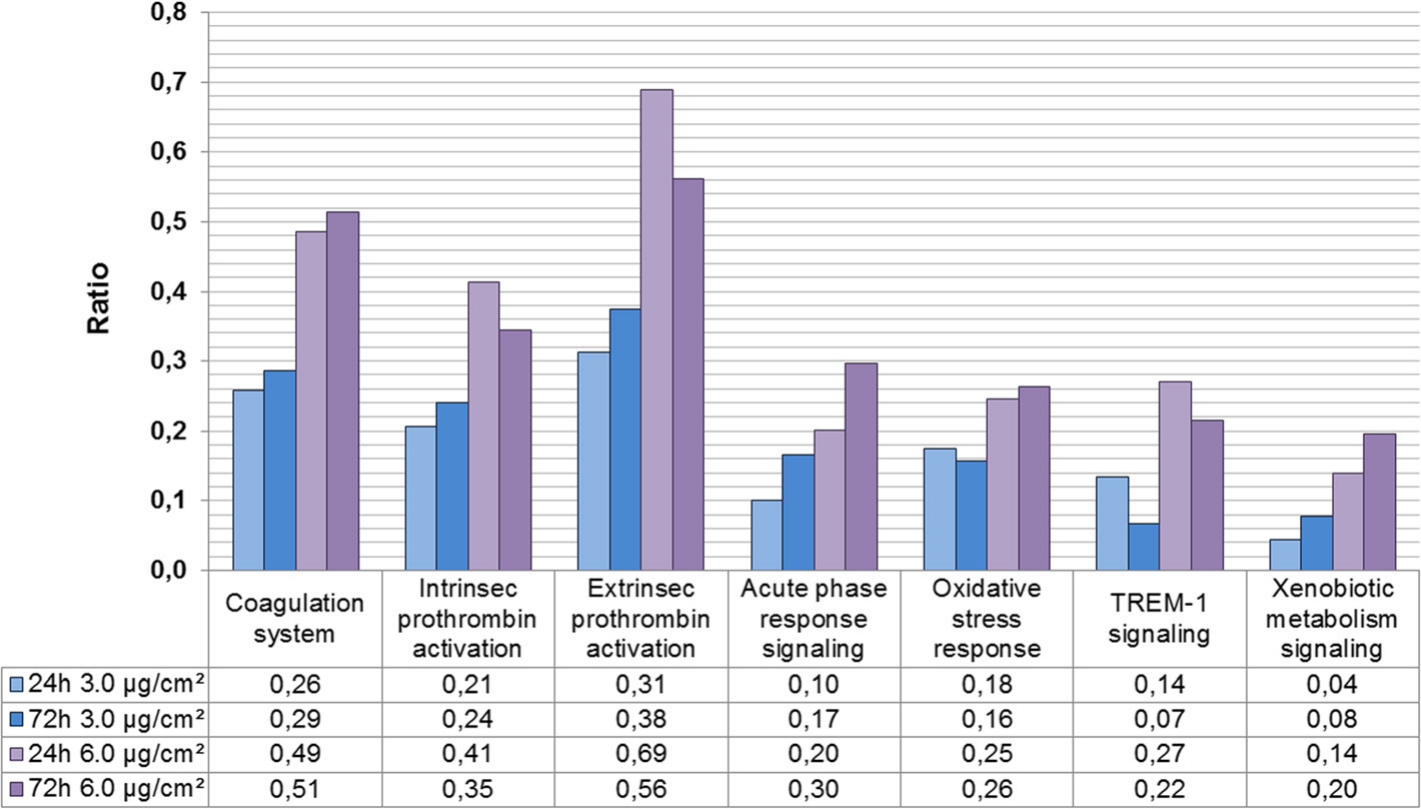
Altered canonical pathways. A549 cells (n = 3) were exposed for 24 h and 72 h to increasing concentrations of fumed silica AEROSIL® 200 NPs (3.0 and 6.0 μg/cm^2^). Seven relevant canonical pathways disturbed after 24 h and 72 h exposure with two doses of NPs (3.0 and 6.0 μg/cm^2^) were revealed with the Ingenuity Pathway Analysis tool. Each canonical pathway is constituted of a finite number of genes. For each dose and time point, we calculated a ratio indicating the percentage of altered genes in our dataset belonging to a given canonical pathway. These pathways are all significant according to a Fisher statistical test (p-value < 0.05).

Figure 6 reports the seven canonical pathways mainly altered by fumed silica NPs in a time- and dose-dependent manner. The most highly altered canonical pathways were related to the “coagulation system”, including “intrinsic” and “extrinsic” “prothrombin activation pathways”.

Other pathways linked to the cell response, and involved at both times and both doses, included “acute phase response signaling”, “xenobiotic metabolism signaling”, “TREM1 signaling” and “oxidative stress response”. Some pathways are not represented in Figure 6 because they were only engaged at the lowest dose (1.5 μg/cm^2^). These pathways are “clathrin-mediated endocytosis”, “Rho signaling pathway” and “actin cytoskeleton remodeling”; they presented low ratios but were obtained with high significance. This allowed specific cellular effects triggered as a function of increasing NP concentrations to be distinguished and to be classified at three hierarchical levels of toxicological response. The Additional file 3: Table S3 indicates more precisely the genes characterizing each pathway for the three levels at all NP concentrations (1.5, 3.0, and 6.0 μg/cm^2^), with their fold changes and p-values.

#### Exoproteomic analysis

We used several tools such as SignalP, SecretomeP, Exocarta, and Vesiclepedia to distinguish the proteins secreted by classical (endoplasmic reticulum/golgi) and non-classical (such as exosomes and microvesicles) export mechanisms from those that were not secreted at 24 h exposure. We then identified their implication in toxicological functions using the IPA database.

Thus, at 3.0 μg/cm^2^, 10 proteins had a signal peptide (score SignalP ≥ 0.9) and were likely secreted by classical mechanisms. In addition, 61 proteins were found in the Exocarta/Vesiclepedia databases and 22 proteins were identified with SecretomeP (without signal peptide and a score ≥ 0.5), representing 64 proteins potentially secreted by non-classical mechanisms. Finally, by appending all databases, it was found that 67/72 proteins were secreted. Remarkably, five proteins were not found with these tools and were considered as possible cellular waste with high half-lives in the extracellular compartment: histone H4 (H4), T-complex protein 1e (TCPE), T-complex protein 1b (TCPB), tubulin b (TBB5) and transitional endoplasmic reticulum ATPase (TERA).

At 6.0 μg/cm^2^, eight proteins had a signal peptide (score SignalP ≥ 0.9) and were secreted by classical mechanisms. At this dose, 71 proteins were found in the Exocarta/Vesiclepedia databases and 26 proteins were identified with SecretomeP (without signal peptide and a score ≥ 0.5), representing a total of 73 proteins potentially secreted by non-classical mechanisms. Finally, by merging all databases, it was found that 74/79 proteins were actually secreted and five proteins would possibly be cellular waste: heterogeneous nuclear ribonucleoproteins A2/B1 (ROA2), transitional endoplasmic reticulum ATPase (TERA), histone H4 (H4), kynureninase (KYNU), and tubulin beta chain (TBB5).

Three predominant toxicological pathways were identified with IPA. The “NRF2-mediated oxidative stress response” was triggered. Among the proteins involved in the NRF2 response, six and seven proteins were identified at 3.0 and 6.0 μg/cm^2^, respectively. The “xenobiotic metabolism signaling” was also involved, with five and six proteins at 3.0 and 6.0 μg/cm^2^, respectively. These proteins include heat-shock proteins (HSP70 and HSP90), detoxification enzymes such as glutathione reductase (GSR), glutathione S-transferase (GSTP1), lactate dehydrogenase (LDHA), peroxiredoxins (PRDX1, PRDX6), thioredoxin reductase (TRXR1), protein disulfide isomerase (PDIA6), and aldo-keto reductases (AKR1B1, AKR1B10, AKR1C1/C2, and AKR1C3). All proteins were upregulated and detected with high sequence coverage (Additional file 2: Table S2).

Among the non-classically secreted proteins, some, initially involved in glycolysis, were upregulated. The list includes enolase 1 (ENO1), glyceraldehyde-3-phosphate (GAPDH), aldolase A (ALDOA), glucose-6-phosphate isomerase (GPI), phosphoglycerate mutase 1 (PGAM1), phosphoglycerate kinase 1 (PGK1), triosephosphate isomerase 1 (TPI1), and pyruvate kinase (PKM). Interestingly, only four proteins were downregulated compared to controls: thrombospondin-1 (TSP-1; −4.5 and −9.0 at 3.0 and 6.0 μg/cm^2^, respectively), transforming growth factor beta-induced (TGFbI; −4.375 at 6.0 μg/cm^2^), agrin (AGRN; −21.0 at 6.0 μg/cm^2^), and nucleobindin 1 (NUCB1; −4.33 at 6.0 μg/cm^2^).

## Discussion

This work evaluated the mode of action of AEROSIL® 200 fumed amorphous silica NPs in lung epithelial cells using complementary omics. We chose this cellular model because inhalation is the major route of exposure for workers in the NP industry. Any toxicity study with nanoparticles requires a thorough characterization of their size. We showed by atomic force microscopy (Figure 1A) that these NPs were disks with average diameter 10 ± 5 nm and thickness 2 nm ± 0.3 nm. We also highlighted the presence of heterogeneous agglomerates (Figure 1B) of average size 160 ± 90 nm. Hydrodynamic diameters of fumed silica NPs were measured by DLS in the presence and absence of fetal bovine serum (FBS) in the culture medium. In the presence of FBS, these NPs were dispersed, with a mean size of 10 ± 4 nm in agreement with the data from the literature [19] and consistent with the data provided by the manufacturer i.e. 12 nm. In the absence of FBS, the silica NPs formed agglomerates whose hydrodynamic diameter reached 350 ± 8 nm. Interestingly, AFM identifies the size and shape of objects whereas DLS provides only their size. Cell exposure was carried out in the absence of serum for two reasons: i) the exoproteome can only be assessed if contaminants from the culture medium and reagents are present in low quantities [16], and ii) attempts to avoid agglomeration by artificial dispersion may lead to underestimation of possible adverse effects [20]. Indeed, interactions between serum proteins and nanoparticles can attenuate or abrogate the potentially harmful effects of NPs [21-23].

### The concept of NOTEL

Previous studies have shown that the number of modulated genes in human cells may be a good indicator of the level of cellular disturbance induced by a xenobiotic (drugs, chemicals, metals, NP) [11,24-27]. The concept of the NOTEL (no observed transcriptional effect level, the concentration at which no effect on the transcriptome is observed) was described by Lobenhofer et al. [13]. Incorporating a genomic endpoint such as the NOTEL, estimated from gene expression data, is of the highest importance in risk assessment [14]. We used conventional viability tests, ATP and XTT (Figure 2), and cell impedance monitoring (xCELLigence) (Figure 3) as preliminary tests to determine suitable NP concentrations and time points for subsequent toxicogenomic studies. With these preliminary techniques, we were thus able to frame the concentrations that were necessary and sufficient to trigger early effects on cellular mechanisms [28] while avoiding excessive cellular damage. Five concentrations (0.1, 1.0, 1.5, 3.0, and 6.0 μg/cm^2^) inducing less than 10% cell death were chosen for transcriptomic experiments. A 24 h exposure was set as the early effect test according to impedance results. The 3.0 and 6.0 μg/cm^2^ doses were also analyzed in gene expression microarrays with an exposure of 72 h (late effects). The secretomes corresponding to cultures exposed to these two concentrations for 24 h were studied by tandem mass spectrometry.

We noted that the NOTEL (1.0 μg/cm^2^) was lower (i.e. more sensitive) than the NOAEL identified with cytotoxicity assays (1.5 μg/cm^2^), as shown in Figure 4. The LOTEL based on transcriptomics was also lower (1.5 μg/cm^2^) than the LOAEL determined from cell viability (3.0 μg/cm^2^). Thus, in our experiment, microarray technology may be considered to be more sensitive compared to viability tests. The NOTEL may be a relevant indicator of biological exposure to quickly benchmark toxic doses of chemicals and mixtures, especially within the framework of large toxicology programs such as REACH [13,14].

### Advantages and pitfalls of omics

The data obtained with our two complementary omics approaches, transcriptomics and exoproteomics, were analyzed using the same bioinformatics tool (IPA), and the biological results were combined resulting in an illustrative example of toxicological integrative analysis. The Venn diagrams showed that more than 80% of genes (Figure 5A) and proteins (Figure 5B) modulated at 3.0 μg/cm^2^ were also listed as modified at 6.0 μg/cm^2^. The two approaches are thus consistent and the results obtained at the two NP doses reinforce the main toxicological findings extracted from the data. The canonical cellular pathways help to understand, in a fast and overall manner, the interactions between the modulated genes and the cellular mechanisms they belong to. The main pathways disturbed by silica NPs are represented in Figure 6. Regulation of gene expression is so finely tuned that two distinct experimental conditions can activate different transcripts (subunits of the same enzymatic complex, early or late molecules and redundant receptors), while belonging to the same pathway governing the same mode of action. Thus, looking at the overall scheme rather than isolated genes allows for a better understanding of cellular mechanisms involved in the response to a toxic substance. *A fortiori*, analyzing the direction of regulation (induction or repression) of each regulated molecule seems pointless in such large eukaryotic systems, and in the state of our current knowledge. Moreover, the mRNAs are produced in an oscillatory manner (in bursts) according to Raj et al. [29]. Their production is likely to obey finely tuned processes that we do not necessarily know. Nevertheless, analyzing a direction of regulation is certainly more significant with multi-dose and multi-time analyses; potentially conserved regulations could therefore be confirmed. This is why, here, we operated microarrays at several times of exposure and doses of fumed silica NPs.

Regarding the analysis of proteins in the culture supernatants, comprehensive exoproteomics is tedious to perform because of the highly diluted nature of secreted proteins present in biological fluids or culture media [30]. Consequently, we had to concentrate the supernatants by precipitation prior to their analysis with a sensitive tandem mass spectrometer. In addition, one cannot exclude the possibility that some proteins may be released in the supernatant by cell death or lysis during the cell culture. This pitfall was minimized by optimizing experimental parameters (confluence maintained at 80%, intensive washing steps prior to exposure and upstream selection of appropriate NP doses) and using specific bioinformatics tools (SignalP, SecretomeP, Exocarta, Vesiclepedia) to distinguish the proteins that were genuinely excreted from those proteins arising from cell lysis. These secreted proteins illustrate the surroundings of the cells and how they interact with their environment.

### Dose-dependent hierarchical response

The hierarchical oxidative stress paradigm, first described by A. Nel and colleagues [28,31], posits that ROS production leads to incremental cellular responses that can be classified as antioxidant defense, proinflammatory effects, and cytotoxicity. According to these authors, in Tier 1, the transcription factor Nrf2 is activated to enhance the expression of phase II enzymes, which attempt to restore redox equilibrium. If the level of oxidant injury increases (Tier 2), cells express proinflammatory cytokines by activating signaling pathways such as the mitogen-activated protein kinase (*MAPK*) and nuclear factor-kappa B (*NF-*κ*B*) cascades. At the highest level of oxidative stress (Tier 3), interference in mitochondrial inner membrane electron transfer or changing the open/closed status of the permeability transition pore could lead to effects on ATP synthesis and the release of proapoptotic factors.

**Figure 7:**
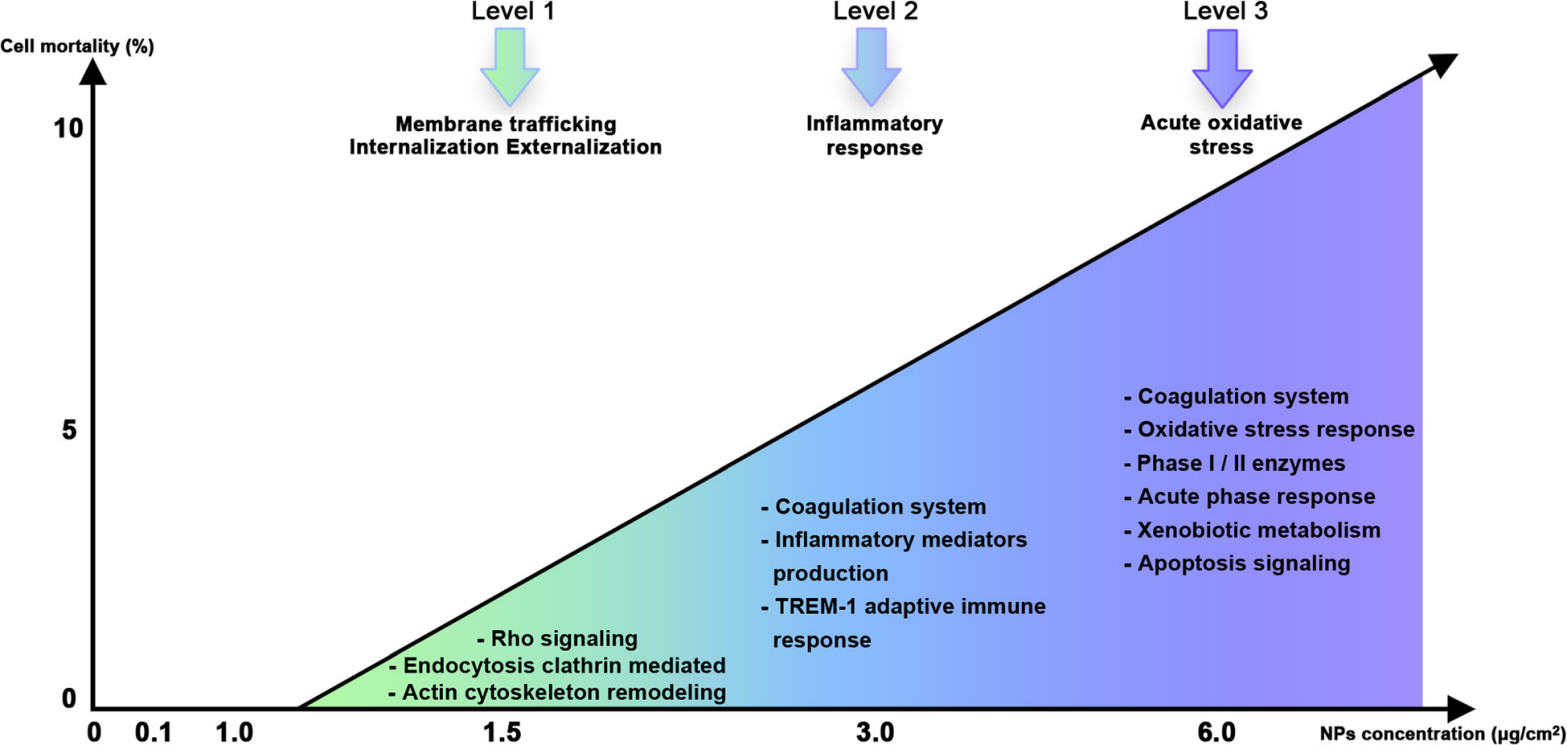
Hierarchical stress response following exposure to increasing concentration of AEROSIL® 200 fumed amorphous silica NPs. A549 cells (n = 3) were exposed for 24 h to increasing concentrations of fumed silica AEROSIL® 200 NPs (0.1, 1.0, 1.5, 3.0 and 6.0 μg/cm^2^). Between 1.0 and 1.5 μg/cm^2^, the silica NPs activated Rho signaling pathways and clathrin-mediated endocytosis. From 3.0 μg/cm^2^, proinflammatory effectors were activated and the “coagulation system pathway” was triggered. Beyond 6.0 μg/cm^2^, the pathways of “xenobiotic metabolism” and “acute phase response” were activated. The vertical scale represents the percentage of dead cells, as determined by ATP assays, in relation to the tested doses of silica NPs. These data exemplify the paradigm of dose dependent toxic events developed by Meng et al. [28]. This figure is freely adapted from their scheme with permission from Meng et al. [28], American Chemical Society, although the quantitative data and pathways are those obtained in experiments described in the current article.

In this study, we illustrated this paradigm to demonstrate that increasingly severe cellular dysfunctions were triggered by silica NPs in a dose-dependent manner (Figure 7). Moreover, our data show cellular mechanisms that occur at lower doses than those that activate oxidative stress.

#### No effect (Level 0)

As depicted in Figure 7, from 0.1 to 1.0 μg fumed silica NPs per cm^2^ cell surface, no effect was detected by transcriptomics. The NOTEL point, the highest concentration of NPs giving no transcriptional effect, was set to 1.0 μg/cm^2^, as two successive values gave no effect.

#### Internalization, externalization (Level 1)

At a very low dose of fumed silica NPs (1.5 μg/cm^2^), we observed an intense signaling activity (Additional file 3: Table S3). Here, we highlighted five induced transcripts belonging to the pathway “clathrin-mediated endocytosis” (*ACTA1, EPS15, HIP1, ORM1 and VEGFA*). This pathway is the major pathway for the internalization of exogenous molecules from the plasma membrane into intracellular compartments. We also observed the induction of downstream effectors of the “Rho signaling pathway” *(ROCK1, ARHGAP24*, *PPP1R12B* and *RAPGEF* group). Rho signaling attests to a cytoskeleton reorganization by stimulating actin dynamics. Rho GTPases regulate membrane trafficking and are important regulators of exocytosis through a process not yet elucidated [32,33].

We did not observe, among the 255 genes differentially expressed at this concentration, any element of antioxidant response. Typically, in our first level, we describe some events that are well below the events described by Nel and colleagues in their Tier 1, i.e. oxidative stress [28,31]. A possible reason may be that antioxidant defense has been shown to be triggered by a higher concentration (>5 μg/cm^2^) [34], and another explanation is that our technology is extremely sensitive in apprehending very subtle events. In our data, the first level rather reflects the endocytosis and exocytosis of NPs, as attested by the cytoskeleton remodeling and membrane trafficking. This likely corresponds to a normal response of cellular defense.

#### Inflammation and nonspecific response (Level 2)

Starting from the 3.0 μg/cm^2^ dose, upregulation of inflammatory mediators (*IL-6*, *IL-8* and *TNF-α*) and chemokines (*CCL5* and *CCL20*) was observed in a dose-dependent manner at 24 h exposure (Additional file 3: Table S3). These results are consistent with those found in the literature for silica and other NPs [4,35,36]. Perkins et al. previously compared crystalline and amorphous silica NPs, but found that only cristobalite upregulated *IL6* and *IL8* in lung BEAS- 2B cells whereas amorphous silica NPs only modulated *CCL5* (RANTES) expression [4]. Here, we observed that not only the above interleukins, but also *CCL5* and *CCL20*, were upregulated in the presence of AEROSIL® 200 fumed silica NPs, with a fold change of 4.5 and 10.2 at 3.0 and 6.0 μg/cm^2^, respectively. This discrepancy between our data and previously published data illustrates the possible heterogeneity of the results in the NP literature, probably directly linked to the different nature of the NPs. In particular, fumed AEROSIL® 200 NPs differ from the amorphous silica NPs of the previous study [4], with a surface area 100 times greater. The “TREM1 signaling pathway” was triggered, leading to proinflammatory immune responses. The *TREM1* transcript, which belongs to the immunoglobulin family of cell surface receptors, was upregulated at 6.0 μg/cm^2^.

#### Coagulation system perturbation as a specific response (Level 2)

At higher concentrations (3.0 and 6.0 μg/cm^2^), and for two exposure durations (24 and 72 h), the canonical “coagulation pathway” was altered by fumed silica NPs (Figure 6). This pathway, and more specifically the “intrinsic and extrinsic prothrombin activation” subgroups, is the most significant altered pathway. Besides the known functions of the alveolar epithelium, such as surfactant secretion (to maintain alveolar stability), gas exchange, and ion transport, these cells also have barrier properties that protect against the passage of air pollutants into the circulation [37]. These authors also demonstrated that in A549 cells, the coagulation process responds to injury by the rapid formation of a clot and fibrin deposition [38].

Our data thus suggest that this pathway is a direct consequence of the alteration of the A549 cell monolayer by fumed silica nanoparticles, probably due to cell membrane alteration. Indeed, as demonstrated by Zhang and colleagues, fumed silica NPs (AEROSIL, diameter 16 nm) induce a dramatically higher cytotoxicity than colloidal silica in lung cells BEAS-2B [27]. The authors explained the increased toxicity of fumed silica by its thermally promoted surface properties, i.e. the formation of strained, three-membered siloxane rings on the silica surface via dehydroxylation. These rings are rapidly hydrolyzed upon rehydration in culture medium, exposing hydroxyl groups responsible for cell toxicity by interaction with cell membranes. In our proteomic data, the cell membrane alteration is confirmed by the release of the LDH protein, which is an indicator of cell membrane integrity.

Coagulation is a complex process that can be initiated via the extrinsic or intrinsic pathways. In the extrinsic pathway, a cascade of activation including factor VIIa (*F7*) and tissue factor (*TF*) leads to the generation of thrombin (*F2*) and the production of fibrin (*FG*), which creates a clot. In the intrinsic pathway, a cascade of activations leads to the production of coagulation factors such as FXa (*F10*). The coagulation system includes a number of control proteins to maintain a fine balance between formation and dissolution of the clot. Plasmin is one of these proteins that are required for dissolution of the fibrin clot. Plasmin is activated by tissue plasminogen activator (*PLAT*) and urokinase (*PLAU*). Protein C, protein S, and thrombomodulin together degrade FVa and FVIIIa.

The modifications of expression of the key actors in the coagulation process are given in Additional file 3: Table S3. *F2* (thrombin), *F7, F10* and *F13b* are downregulated, as are *FGA, FGB* and *FGG* (fibrinogen chains), and *KLK1* (kallikrein 1). *THBD* (thrombomodulin) is upregulated, likely reducing coagulation and activating the anticoagulant pathway. *PROC*, *PROS1, and TFP1* (lipoprotein-associated coagulation inhibitor) are also differentially expressed in our datasets*.* In addition, *PLAT (*tissue plasminogen activator*), PLAU* (urokinase), and *PLAUR* are upregulated. These participate in the conversion of plasminogen to plasmin, thus activating fibrinolysis to dissolve fibrin clots.

**Figure 8:**
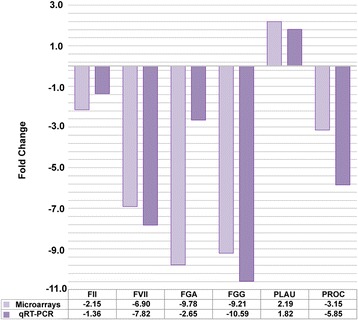
Changes in the mRNA expression measured by transcriptome and by real-time PCR. The fold changes of *FGA, F2, PLAU, FGG, F7*, and *PROC* in cells treated with fumed silica AEROSIL® 200 NPs (6.0 μg/cm^2^ at 72 h) are expressed versus untreated cells. For qRT-PCR (n = 6) normalization was based on the total RNA mass quantified on the Nanodrop. All expressions in treated cells were significantly different from controls at p < 0.05. These genes are all connected to the “coagulation pathway” and the qRT-PCR findings confirm transcriptomic data.

Although the interpretation of the direction of the regulation (up or down) of each individual molecular component of a pathway is far from being straightforward in such a complex system, it is interesting to note that key molecules of the coagulation system have a conserved modulation with both dose and time of exposure. The modifications in expression of the main transcripts of the coagulation system were confirmed by qRT-PCR (Figure 8).

Hence it is difficult to determine if these genes activate wound healing by fibrinogenesis or if, on the contrary, they destroy fibrin clots by fibrinolysis. It is likely that both mechanisms are at work, as the coagulation system responds to injury through a cascade of events leading to the formation of a clot that is eventually degraded and resorbed through the process of fibrinolysis. It is possible that, at 24 h and 72 h after exposure, only the final stage of the process is detectable. Proteomics revealed the inhibition of thrombospondin (TSP-1) which participates in the plasminogen activation and fibrinolysis [39].

According to Napierska et al., inhaled particles are involved in a procoagulant response in lung models [40]. Available data from experimental instillation/inhalation of engineered nanoparticles are supportive of the hypothesis that a pulmonary and/or systemic inflammatory response induces a procoagulatory state [41,42]. Therefore, in coherence with the literature, our data support an effect of these silica nanoparticles on the coagulation system, and argue for the involvement of this specific mechanism starting at level 2.

#### Toxic oxidative stress (Level 3)

At the 6.0 μg/cm^2^ dose, we observed by transcriptomics the first signs of oxidative stress (Additional file 3: Table S3), caused by an imbalance between the production of reactive oxygen and the detoxification of reactive intermediates, especially the NRF2 signaling cascade, antioxidant molecules (*SOD, GPX2*), and phase I and phase II enzymes (*GST, EPHX1,GCLC and AOX1*). Proteomics clearly indicated the presence of the “NRF2-mediated oxidative stress response”, with the induction of the enzymes, PRDX1(R = 6.4) and TXNRD1(R = 13).

Our transcriptomics results highlighted the “acute phase response signaling” pathway, a nonspecific defense mechanism. This pathway is triggered by tissue injury and characterized by the upregulation of proinflammatory cytokines and acute phase proteins such as *NFKB2*, *IL6,* and *SOD2* at 3.0 and 6.0 μg/cm^2^. “Xenobiotic metabolism signaling” is also activated, with several transcripts encoding enzymes such as *ALDHA*, *MAP2K3*, and *TNF* at 6.0 μg/cm^2^. Proteomics confirmed this trend with the overexpression of aldehyde dehydrogenase ALDHA (FC =20.4 and 21.5), glutathione transferase GSTP1 (FC = 11.0 and 11.3), and heat-shock protein HSP90A (FC = 14.2 and 19.4), at 3.0 and 6.0 μg/cm^2^, respectively.

### Toxicogenomics and risk assessment

The hierarchical response of alveolar epithelial lung cells (Figure 7) illustrates quantitatively the perspectives developed by A. Nel and colleagues [28,31,43] regarding the safety assessment of nanomaterials.

The genome-wide response to nanomaterial exposure remains largely unexplored and the use of omics technologies in the safety assessment of nanomaterials would be interesting for predictive toxicology [25]. We have demonstrated that toxicogenomics can provide a quantitative assessment of toxicity and, at the same time, gives information about the mode of action of toxicants, possibly revealing adverse outcome pathways (AOPs). In their vision and strategy for toxicity testing in the 21^st^ century, the National Research Council cited the linkage of adverse effects to specific toxicity pathways and “Applications of Toxicogenomic Technologies to Predictive Toxicology and Risk Assessment”. Recently, EFSA delivered a scientific report recommending for future work and research the use of omics technologies and AOPs in the context of the prioritization of chemicals for risk assessment [44].

In our study, the use of these two techniques allowed us to establish a precise dose–response curve of fumed silica NPs, with a NOTEL, and to link these doses to specific pathways, allowing insights into mechanistic information for both intracellular and extracellular compartments.

## Conclusions

Transcriptomic and proteomic approaches were combined in the current study to investigate quantitatively the effects of AEROSIL® 200 fumed silica NPs in A549 epithelial lung cells, and within their close environment to decipher their mode of action. Such an alliance of omics has rarely been reported regarding the toxicological effects of NPs. Our main findings allowed the setting of a NOTEL (no observed transcriptional effect level) for fumed silica NPs at 1.0 μg/cm^2^, a threshold lower than values previously obtained with conventional viability tests. Moreover, the NOTEL may be a relevant indicator of biological exposure for use in quickly benchmarking doses of chemicals and mixtures in toxicology.

These techniques allowed us to establish a hierarchical dose–response curve of fumed silica NPs, linking doses to adverse outcome pathways, and delivering not only quantified cytotoxic values but also mechanistic information for both the intracellular and extracellular compartments. Monitoring the overall effects rather than those of isolated genes/proteins thus allows for a better understanding of the cellular mechanisms involved in the response to a toxic substance, without losing the possibility to analyze very specific molecular effects. As in photography, toxicogenomics allows, at the same time, the acquisition of a wide vision of biological responses and a “zoom” into the details with a great depth of field. Indeed the hypotheses generated by these results regarding a specific adverse effect can be studied further with more focused techniques. This approach might be adapted to any type of potentially toxic substance, alone or in a mixture, in cellular models as a predictive tool for toxicity assessment in a future regulatory perspective.

## Methods

### Nanoparticle characterization

AEROSIL® 200 particles (hydrophilic fumed silica, from Evonik, Germany) are extrapure (SiO_2_ > 99.8%), nanosized (12 nm diameter), and have a high specific surface area of 200 m^2^/g, according to the supplier. These nanoparticles were used “as received”. The aggregation states of the nanoparticles were analyzed by dynamic light scattering (n = 3) in the culture medium, with/without serum, using the Zetasizer Nano ZS (Malvern Instruments Ltd, UK). Atomic force microscopy imaging was performed with a Multimode and Nanoscope V instrument (Veeco, Santa Barbara, CA, USA) mounted with an EV Scanner and silicium tips (*K* = 0.58 N/nm, Bruker). Sample preparation consisted of the deposition of 5 μl SiO_2_ NP suspension (at 10 μg/ml in pure water) on freshly cleaved Mica (Muscovite from SPI, France). After 1 min., the sample was dried under vacuum. Amplitude and height images were recorded in peak force mode. The resulting pictures were plane-fitted and flattened with the supplied Nanoscope Analysis software (Brucker) V 1.40.

### Cell culture

The A549 human alveolar epithelial cell line (CCL −185™) was obtained from the American Type Culture Collection (ATCC, Manassas, VA, USA). Cells were cultured in DMEM/F12 medium (Invitrogen) supplemented with 10% fetal bovine serum (FBS) and 1% penicillin/streptomycin (100 U/mL, 100 μg/mL) and incubated in a humidified incubator at 37°C and 5% CO_2_. Cells were used between passages 20 to 40. Cells were passed twice a week and the medium was changed three times per week, keeping confluence below 80%.

### Cell viability tests

*ATP tests.* A549 cells were grown in 96-well plates. The cells were exposed for 24 h at serial dilutions of SiO_2_ NPs in serum-free medium (from 0.3 μg/cm^2^ to 30.3 μg/cm^2^ of cell surface). Cells were washed with PBS and cell viability was determined by the ATP test (Cell Titer-Glo luminescent cell viability Assay), as specified by the supplier (Promega). Briefly, 100 μl kit reagent were added per well and the plate was shaken for 10 min. at RT before measuring bioluminescence with a LUMIstar Galaxy instrument (BMG).

*XTT tests.* A549 cells were grown in 96-well plates. The cells were exposed for 24 h at serial dilutions of SiO_2_ NPs in serum-free medium. Cells were washed with PBS and cell viability was determined by the XTT test (Sigma-TOX2, *In Vitro* Toxicology Assay Kit, XTT based), as recommended by the supplier (Sigma-Aldrich). Briefly, 20 μl kit reagent were added per well and the plate was incubated for 2 h at 37°C before reading the absorbance at 450 nm and 690 nm with a Multiscan Spectrum spectrophotometer (Thermo Electron Corporation).

### Real-time impedance measurement (Xcelligence)

A background resistance of the E-plates (ACEA) was determined with 100 μl culture medium. A549 cells were seeded in E-plates at 10,000 cells in 100 μL. E-plates were placed into the Real-Time Cell Analyzer (RTCA) station (ACEA) and incubated at 37°C. Cells were grown for 24 h, with impedance measured every minute during 14 h (adhesion phase), then every 15 min. (growth phase) for 14 h. After 28 h, cells were exposed (n = 3) to SiO_2_ NPs at 1.5, 3.0, 6.0, 25, and 50 μg/cm^2^, and were monitored every minute during 12 h (early effects) then every 15 min. for 100 h (late effects). The impedance of control cells without SiO_2_ NPs was also recorded. Cell index (CI) raw data values were calculated by the apparatus software (RTCA software 2.0). Normalized cell indexes were also calculated by the software at a selected normalization time point, which was chosen as the time just before the addition of nanoparticles.

### Microarrays (Agilent Human V2 4x44K)

A549 cultures (biological triplicate) were exposed to five concentrations of SiO_2_ NPs (0.1, 1.0, 1.5, 3.0, and 6.0 μg/cm^2^) and serum-free medium (control cells) for 24 h, and exposed to two concentrations of SiO_2_ NPs (3.0 and 6.0 μg/cm^2^) for 72 h. Total RNAs were extracted using the RNeasy Mini Kit (Qiagen). RNAs were quantified with the NanoDrop 1000 spectrophotometer and their qualities were assessed with the Agilent 2100 Bioanalyzer (Agilent). RNA samples were amplified and labeled with cyanine-3 fluorophore using a Low Input Quick Amp Labeling Kit (Agilent). Hybridization (n = 3) was performed with Human V2 4x44K oligo microarrays (Agilent). Fluorescence was scanned (Agilent Scanner) and signal data were extracted with Feature Extraction software (Agilent).

### Statistical analysis for transcriptomics

Five independent analyses were conducted, namely SiO_2_ NP-exposed cells versus control cells at five different concentrations: 0.1, 1.0, 1.5, 3.0, and 6.0 μg/cm^2^. For each analysis, six fluorescence data files (three for exposed cells and three for control cells) were submitted to GeneSpring GX 11 software (Agilent Technologies) using a widespread method for determining the significance change of gene expression [45,46]. The fold change cutoff between control and exposed samples was set to 1.5. Genes significantly up- or downregulated were determined by an unpaired t-test with a p-value below 0.05 and a Benjamini-Hochberg false discovery rate correction. We thus obtained lists of genes that were significantly induced or repressed after exposure to NPs.

### qRT-PCR validation of microarray data focused on coagulation

Validation of whole-genome expression microarray was performed by qRT-PCR on a representative list of significant genes. Total RNA was isolated according to the manufacturer's instructions using the RNeasy kit (Qiagen) and treated with DNase. RNA purity and concentration were determined by UV on a Nanodrop® Spectrophotometer and integrity was assessed on an Agilent 2100 Bioanalyzer (Agilent Technologies). Differential analysis of transcripts from cells exposed to 6.0 μg/cm^2^ SiO_2_ NPs for 72 h versus unexposed cells was performed by qRT-PCR on Opticon II (Biorad) with the Sybr Green PCR Master Mix (Finzyme) kit according to the manufacturer's instructions. Measurements were means of sextuplets (duplicates of three independent experiments). Primer sequences are listed in the Additional file 4: Table S4.

### Sample preparation for shotgun proteomics and tandem mass spectrometry

Culture supernatants of control cells and those exposed to fumed silica NPs during 24 h were collected and pooled (v = 2.5 mL per condition, biological triplicates). Proteins from each sample were precipitated with 10% trichloroacetic acid after the addition of a one-quarter volume of trichloroacetic acid at 50% (w/vol). The solutions were vortexed and then incubated on ice for 10 min. The samples were centrifuged at 16,000 g for 10 min. at 4°C. The resulting pellet was dissolved in 45 μl 1X LDS (Invitrogen). These samples were heated at 99°C for 5 min. and then loaded onto a 10% NuPAGE gel (Invitrogen) for a short electrophoresis in MOPS buffer. The polyacrylamide bands containing the whole exoproteomes were processed as previously described [47]. The resulting peptides (10 μl of the 40 μl generated with the procedure) were analyzed with an LTQ-Orbitrap XL hybrid mass spectrometer (ThermoFisher) coupled to an UltiMate 3000 Nano LC System (Dionex-LC Packings), as described previously [48].

### Protein identification and quantification by shotgun proteomics

The MS/MS spectra were searched against the SwissProt database (release SwissProt_2013_01.fasta) totaling 65,754 protein sequences from mammals. Searches for trypsic peptides were performed with the following parameters: full-trypsin specificity, a mass tolerance of 5 ppm on the parent ion and 0.5 Da on the MS/MS, carbamidomethyl Cys as static modification and oxidized Met as dynamic modification, and maximum number of missed cleavages set at 2. All peptide matches with a peptide score below a p-value of 0.05 were filtered. A protein was considered to be validated when at least two different peptides were detected in the same experiment. The false-positive rate for protein identification was estimated using the appropriate decoy database as below 1%. The number of MS/MS spectra per protein (spectral counts) was determined for each sample. Spectral counts were then compared using the TFold method of the PatternLab software [49]. Fold change and p-value cutoffs were set at 2.0 and 0.05, respectively.

### Integrative biological analysis

Cellular function and localization of identified genes and proteins were obtained with the Ingenuity Pathway Analysis software (Ingenuity Systems, US).

Lists of genes or proteins significantly induced or repressed after exposure to NPs were uploaded into Ingenuity Pathway Analysis software for biological analysis by comparison with the Ingenuity Knowledge Database. These lists of altered genes or proteins were then processed to investigate their functional distribution, as defined by Gene Ontology. Datasets were analyzed with several tools such as SecretomeP, SignalP, Exocarta and Vesiclepedia. Known canonical pathway associations were measured by IPA by using a ratio of the number of genes or proteins from datasets that map to the pathway divided by the total number of constitutive genes or proteins that map to the canonical pathway. A Fisher’s exact test was used to determine a p-value representing the significance of these associations.

### Availability of supporting data section

The data sets supporting the results of this article are available as Additional files. The raw data discussed in this publication have been deposited in the NCBI Gene Expression Omnibus (GEO) repository and are accessible through GEO Series accession number (http://www.ncbi.nlm.nih.gov/geo/query/acc.cgi?acc=GSE63806).

## Additional files

Additional file 1: Table S1. List of genes significantly differentially expressed upon exposure to AEROSIL® 200 fumed amorphous silica NPs versus control cells. Three different A549 cultures were exposed to five concentrations of SiO_2_ NPs (0.1, 1.0, 1.5, 3.0, and 6.0 μg/cm^2^) and serum-free medium (control cells) for 24 h, and to two concentrations (3.0 and 6.0 μg/cm^2^) for 72 h. Hybridization (n = 3) was performed with Human V2 4x44K oligo microarrays (Agilent). The fold change cutoff between control and exposed samples was set to 1.5. Genes significantly up- or downregulated were determined by an unpaired t-test with a p-value below 0.05 and a Benjamini-Hochberg false discovery rate (n = 3). Additional file 2: Table S2. List of proteins identified in the exoproteome by nanoLC-MS/MS. Spectral counts per protein measured for each of the nine samples; control (Ctrl), first concentration (C1), and second concentration (C2), with three replicates, are indicated, as well as the PatternLab statistical results (fold change, p-value, signal +, signal -, class) for the three possible comparisons (C1 versus Crtl; C2 versus Crtl; C2 versus C1). The patternLab classes are: blue (identifications that satisfied both the fold and statistical criteria), orange (identifications that did not meet the fold criterion but have low p-values), green (identifications that satisfied the fold criteria but this was most likely to have happened by chance), and red (identifications that did not meet the fold and p-value criteria). Additional file 3: Table S3. List of genes and proteins supporting the hierarchical stress response. A549 cells were exposed to 1.5, 3.0, and 6.0 μg/cm^2^ silica NPs for 24 h. Fold changes of genes and proteins significantly up- or downregulated were determined by an unpaired t-test (p < 0.05) and a false discovery rate correction. Gene symbols are indicated in italics, protein symbols are not italicized. * Transcripts whose expression is identical in control cells and in NPs-exposed cells. Additional file 4: Table S4. List of primers used in qRT-PCR. Differential analysis of transcripts from cells exposed to 6.0 μg/cm^2^ SiO_2_ NPs for 72 h versus unexposed cells was performed by qRT-PCR with the Sybr Green PCR Master Mix (Finzyme) kit according to the manufacturer's instructions on Opticon II (Biorad). This pool of genes represents the “coagulation system” pathway.
