# Supplementary material for: High-throughput, quantitative assessment of the effects of low-dose silica nanoparticles on lung cells: grasping complex toxicity with a great depth of field
Source: BMC Genomics. 2015 Apr 18;16(1):315. doi: 10.1186/s12864-015-1521-5 (PMC4404697; doi:10.1186/s12864-015-1521-5)
Supplement: Additional file 3: Table S3. — List of genes and proteins supporting the hierarchical stress response. A549 cells were exposed to 1.5, 3.0, and 6.0 μg/cm2 silica NPs for 24 h. Fold changes of genes and proteins significantly up- or downregulated were determined by an unpaired t-test (p < 0.05) and a false discovery rate correction. Gene symbols are indicated in italics, protein symbols are not italicized. * Transcripts whose expression is identical in control cells and in NPs-exposed cells. [file 12864_2015_1521_MOESM3_ESM.docx]

**Table S3: List of transcripts representative of stress-response pathways.**

|  | **Transcriptomic response Level 1** | | |  |  |  |
| --- | --- | --- | --- | --- | --- | --- |
|  | |  |  | |  |  |
| ***Symbol*** | | **Entrez Gene Name** | **Fold Change**  **(1,5 µg/cm²)** | | **Fold Change (3,0 µg/cm²)** | **Fold Change**  **(6,0 µg/cm²)** |
| ***Rho signaling and actin cytoskeleton signaling*** | | |  | |  |  |
| *ACTA1* | | actin, alpha 1, skeletal muscle | 1,7 | | *** | *** |
| *ARHGAP24* | | Rho GTPase activating protein 24 | 2,0 | | *** | *** |
| *PPP1R12B* | | protein phosphatase 1, regulatory subunit 12B | 2,7 | | *** | *** |
| *RAPGEF2* | | Rap guanine nucleotide exchange factor (GEF) 2 | 1,7 | | *** | *** |
| *RAPGEF6* | | Rap guanine nucleotide exchange factor (GEF) 6 | 1,7 | | *** | *** |
| *ROCK1* | | Rho-associated, coiled-coil containing protein kinase 1 | 1,8 | | *** | *** |
|  | |  |  | |  |  |
| ***Clathrin-mediated endocytose*** | | |  | |  |  |
| *ACTA1* | | actin, alpha 1, skeletal muscle | 1,7 | | *** | *** |
| *EPS15* | | epidermal growth factor receptor pathway substrate 15 | 2,4 | | *** | *** |
| *HIP1* | | huntingtin interacting protein 1 | 1,5 | | *** | *** |
| *ORM1* | | orosomucoid 1 | 1,7 | | *** | *** |
| *VEGFA* | | vascular endothelial growth factor A | 1,5 | | *** | *** |
|  | |  |  | |  |  |
|  | |  |  | |  |  |
|  | | **Transcriptomic Level 2 and Level 3** | | |  |  |
| ***Main inflammatory effectors*** | | |  | |  |  |
| *IL-11* | | Interleukin 11 | *** | | 1.80 | 2.27 |
| *CCL20* | | chemokine (C-C motif) ligand 20 | *** | | 1.79 | 3.41 |
| *CCL5* | | chemokine (C-C motif) ligand 5 | *** | | 4.50 | 10.14 |
| *CSF2* | | colony stimulating factor 2 (granulocyte-macrophage) | *** | | 2,865 | 5,05 |
| *CSF3* | | colony stimulating factor 3 (granulocyte) | *** | | *** | 7,271 |
| *IL-23alpha* | | Interleukin 23, alpha subunit p29 | *** | | 1.97 | 2.81 |
| *IL-24* | | Interleukin 24 | *** | | 4.39 | 24.68 |
| *IL-32* | | Interleukin 32 | *** | | 1.56 | 2.22 |
| *IL-4i1* | | Interleukin 4 induced 1 | *** | | 2.93 | 4.45 |
| *IL-6* | | Interleukin 6 | *** | | 4.20 | 13.51 |
| *IL-8* | | Interleukin 8 | *** | | *** | 2.36 |
| *NFKB2* | | nuclear factor of kappa light polypeptide gene enhancer in B-cells 2 (p49/p100) | *** | | 2,038 | 3,043 |
| *SERP1* | | stress-associated endoplasmic reticulum protein 1 | *** | | -2,404 | *** |
| *TGFalpha* | | Transforming growth factor, alpha | *** | | 1.58 | 2.09 |
| *TNFalpha* | | Tumor necrosis factor | *** | | *** | 3.51 |
| ***Coagulation system*** | | |  | |  |  |
| *BDKRB1* | | bradykinin receptor B1 | *** | | -2,0 | -1,8 |
| *BDKRB2* | | bradykinin receptor B2 | *** | | -2,1 | -2,2 |
| *COL10A1* | | collagen, type X, alpha 1 | *** | | * | -1,6 |
| *COL1A1* | | collagen, type I, alpha 1 | *** | | -2,4 | -3,6 |
| *F10* | | coagulation factor X | *** | | * | -2,4 |
| *F13B* | | coagulation factor XIII, B polypeptide | *** | | * | -3,5 |
| *F2* | | coagulation factor II (thrombin) | *** | | * | -1,7 |
| *F2R* | | coagulation factor II (thrombin) receptor | *** | | * | 1,5 |
| *F7* | | coagulation factor VII (serum prothrombin conversion accelerator) | *** | | -2,5 | -4,4 |
| *FGA* | | fibrinogen alpha chain | *** | | -1,9 | -3,1 |
| *FGB* | | fibrinogen beta chain | *** | | -2,1 | -3,3 |
| *FGG* | | fibrinogen gamma chain | *** | | -2,1 | -3,1 |
| *KLK1* | | kallikrein 1 | *** | | -1,8 | -2,1 |
| *PLAT* | | plasminogen activator, tissue | *** | | * | 1,8 |
| *PLAU* | | plasminogen activator, urokinase | *** | | 1,7 | 2,2 |
| *PLAUR* | | plasminogen activator, urokinase receptor | *** | | 1,6 | 2,2 |
| *PROC* | | protein C (inactivator of coagulation factors Va and VIIIa) | *** | | -2,0 | -3,0 |
| *PROS1* | | protein S (alpha) | *** | | * | -1,6 |
| *TFPI* | | tissue factor pathway inhibitor (lipoprotein-associated coagulation inhibitor) | *** | | * | 2,1 |
| *THBD* | | thrombomodulin | *** | | * | 1,6 |
|  | |  |  | |  |  |
| ***Acute phase response signaling*** | | |  | |  |  |
| *AHSG* | | alpha-2-HS-glycoprotein | *** | | * | -1,9 |
| *ALB* | | albumin | *** | | * | -3,6 |
| *AMBP* | | alpha-1-microglobulin/bikunin precursor | *** | | -2,0 | -3,0 |
| *APOH* | | apolipoprotein H (beta-2-glycoprotein I) | *** | | -2,1 | -4,0 |
| *C4BPA* | | complement component 4 binding protein, alpha | *** | | -1,9 | -2,7 |
| *C5* | | complement component 5 | *** | | * | -1,9 |
| *CEBPB* | | CCAAT/enhancer binding protein (C/EBP), beta | *** | | * | 1,7 |
| *CP* | | ceruloplasmin (ferroxidase) | *** | | -1,7 | -1,5 |
| *F2* | | coagulation factor II (thrombin) | *** | | * | -1,7 |
| *FGA* | | fibrinogen alpha chain | *** | | -1,9 | -3,1 |
| *FGB* | | fibrinogen beta chain | *** | | -2,1 | -3,3 |
| *FGG* | | fibrinogen gamma chain | *** | | -2,1 | -3,1 |
| *FN1* | | fibronectin 1 | *** | | * | -1,6 |
| *FOS* | | FBJ murine osteosarcoma viral oncogene homolog | *** | | -1,6 | -1,6 |
| *HNF1A* | | HNF1 homeobox A | *** | | * | -1,7 |
| *HSPA1A/HSPA1B* | | heat shock 70kDa protein 1A | *** | | * | 2,2 |
| *HSPA6* | | heat shock 70kDa protein 6 (HSP70B') | *** | | 5,9 | 25,2 |
| *HSPB11* | | heat shock protein family B (small), member 11 | *** | | 2,7 | * |
| *HSPB8* | | heat shock 22kDa protein 8 | *** | | 1,5 | 2,5 |
| *HSPB9* | | heat shock protein, alpha-crystallin-related, B9 | *** | | * | -1,5 |
| *HSPBAP1* | | HSPB (heat shock 27kDa) associated protein 1 | *** | | * | 1,5 |
| *IL6* | | interleukin 6 | *** | | 4,2 | 13,5 |
| *ITIH2* | | inter-alpha-trypsin inhibitor heavy chain 2 | *** | | * | -2,0 |
| *MAP2K3* | | mitogen-activated protein kinase kinase 3 | *** | | * | 1,7 |
| *MAP2K6* | | mitogen-activated protein kinase kinase 6 | *** | | * | -1,6 |
| *MAP3K1* | | mitogen-activated protein kinase kinase kinase 1, E3 ubiquitin protein ligase | *** | | * | -1,5 |
| *NFKB2* | | nuclear factor of kappa light polypeptide gene enhancer in B-cells 2 (p49/p100) | *** | | 2,0 | 3,0 |
| *NFKBIA* | | nuclear factor of kappa light polypeptide gene enhancer in B-cells inhibitor, alpha | *** | | * | 1,6 |
| *NFKBIB* | | nuclear factor of kappa light polypeptide gene enhancer in B-cells inhibitor, beta | *** | | * | 1,5 |
| *NFKBIE* | | nuclear factor of kappa light polypeptide gene enhancer in B-cells inhibitor, epsilon | *** | | * | 1,8 |
| *OSMR* | | oncostatin M receptor | *** | | * | 1,8 |
| *RBP4* | | retinol binding protein 4, plasma | *** | | * | -1,7 |
| *RBP5* | | retinol binding protein 5, cellular | *** | | * | 2,7 |
| *SAA1* | | serum amyloid A1 | *** | | 2,3 | 3,4 |
| *SAA2* | | serum amyloid A2 | *** | | 3,4 | 5,7 |
| *SERPINF1* | | serpin peptidase inhibitor, clade F (alpha-2 antiplasmin, pigment epithelium derived factor), member 1 | *** | | -2,2 | -2,8 |
| *SOCS2* | | suppressor of cytokine signaling 2 | *** | | 1,6 | 1,9 |
| *SOD2* | | superoxide dismutase 2, mitochondrial | *** | | 2,2 | 3,7 |
| *TNF* | | tumor necrosis factor | *** | | * | 3,5 |
| *TNFRSF11B* | | tumor necrosis factor receptor superfamily, member 11b | *** | | * | -2,1 |
|  | |  |  | |  |  |
| ***Oxidative stress response including NRF2-mediated stress response*** | | | | |  |  |
| *ACTA2* | | actin, alpha 2, smooth muscle, aorta | *** | | * | -1,6 |
| *AOX1* | | aldehyde oxidase 1 | *** | | * | 1,9 |
| *DNAJA4* | | DnaJ (Hsp40) homolog, subfamily A, member 4 | *** | | * | 2,1 |
| *DNAJB9* | | DnaJ (Hsp40) homolog, subfamily B, member 9 | *** | | * | 1,7 |
| *EPHX1* | | epoxide hydrolase 1, microsomal (xenobiotic) | *** | | * | -1,6 |
| *FKBP5* | | FK506 binding protein 5 | *** | | * | -1,5 |
| *FOS* | | FBJ murine osteosarcoma viral oncogene homolog | *** | | -1,6 | -1,6 |
| *FOSL1* | | FOS-like antigen 1 | *** | | 1,6 | 2,4 |
| *GCLC* | | glutamate-cysteine ligase, catalytic subunit | *** | | * | -1,9 |
| *GPX2* | | glutathione peroxidase 2 (gastrointestinal) | *** | | -1,7 | -2,5 |
| *GSTA2* | | glutathione S-transferase alpha 2 | *** | | * | -1,7 |
| *GSTA4* | | glutathione S-transferase alpha 4 | *** | | * | -1,6 |
| *GSTA5* | | glutathione S-transferase alpha 5 | *** | | -6,3 | -6,5 |
| *HSPB8* | | heat shock 22kDa protein 8 | *** | | 1,5 | 2,5 |
| *MAFF* | | v-maf avian musculoaponeurotic fibrosarcoma oncogene homolog F | *** | | 1,8 | 2,9 |
| *MAFK* | | v-maf avian musculoaponeurotic fibrosarcoma oncogene homolog K | *** | | * | 1,7 |
| *MAP2K3* | | mitogen-activated protein kinase kinase 3 | *** | | * | 1,7 |
| *MAP2K6* | | mitogen-activated protein kinase kinase 6 | *** | | * | -1,6 |
| *MAP3K1* | | mitogen-activated protein kinase kinase kinase 1, E3 ubiquitin protein ligase | *** | | * | -1,5 |
| *PRKCE* | | protein kinase C, epsilon | *** | | * | 1,7 |
| *SOD2* | | superoxide dismutase 2, mitochondrial | *** | | 2,2 | 3,7 |
| *SQSTM1* | | sequestosome 1 | *** | | * | 1,8 |
|  | |  |  | |  |  |
| ***Xenobiotic Metabolism Signaling*** | | |  | |  |  |
| *ALDH1A3* | | aldehyde dehydrogenase 1 family, member A3 | *** | | * | 1,8 |
| *ALDH1L1* | | aldehyde dehydrogenase 1 family, member L1 | *** | | * | -3,1 |
| *ALDH3A2* | | aldehyde dehydrogenase 3 family, member A2 | *** | | * | -1,5 |
| *CES1* | | carboxylesterase 1 | *** | | * | -1,6 |
| *CHST2* | | carbohydrate (N-acetylglucosamine-6-O) sulfotransferase 2 | *** | | 2,6 | 4,2 |
| *CITED2* | | Cbp/p300-interacting transactivator, with Glu/Asp-rich carboxy-terminal domain, 2 | *** | | * | 2,3 |
| *CYP2C8* | | cytochrome P450, family 2, subfamily C, polypeptide 8 | *** | | * | -2,0 |
| *CYP3A5* | | cytochrome P450, family 3, subfamily A, polypeptide 5 | *** | | * | -1,6 |
| *CYP3A7* | | cytochrome P450, family 3, subfamily A, polypeptide 7 | *** | | * | -1,7 |
| *FMO5* | | flavin containing monooxygenase 5 | *** | | -3,9 | -4,2 |
| *GCLC* | | glutamate-cysteine ligase, catalytic subunit | *** | | * | -1,9 |
| *GSTA2* | | glutathione S-transferase alpha 2 | *** | | * | -1,7 |
| *GSTA4* | | glutathione S-transferase alpha 4 | *** | | * | -1,6 |
| *GSTA5* | | glutathione S-transferase alpha 5 | *** | | -6,3 | -6,5 |
| *IL6* | | interleukin 6 | *** | | 4,2 | 13,5 |
| *IL4I1* | | interleukin 4 induced 1 | *** | | 2,9 | 4,4 |
| *MAOA* | | monoamine oxidase A | *** | | * | -1,5 |
| *MAOB* | | monoamine oxidase B | *** | | * | -1,8 |
| *MAP2K3* | | mitogen-activated protein kinase kinase 3 | *** | | * | 1,7 |
| *MAP2K6* | | mitogen-activated protein kinase kinase 6 | *** | | * | -1,6 |
| *MAP3K1* | | mitogen-activated protein kinase kinase kinase 1, E3 ubiquitin protein ligase | *** | | * | -1,5 |
| *MAP3K10* | | mitogen-activated protein kinase kinase kinase 10 | *** | | * | 1,7 |
| *NDST4* | | N-deacetylase/N-sulfotransferase (heparan glucosaminyl) 4 | *** | | -2,5 | -2,6 |
| *NFKB2* | | nuclear factor of kappa light polypeptide gene enhancer in B-cells 2 (p49/p100) | *** | | 2,0 | 3,0 |
| *PRKCE* | | protein kinase C, epsilon | *** | | * | 1,7 |
| *SMOX* | | spermine oxidase | *** | | 1,9 | 3,2 |
| *SULT1C2* | | sulfotransferase family, cytosolic, 1C, member 2 | *** | | 2,1 | 2,7 |
| *SULT1C3* | | sulfotransferase family, cytosolic, 1C, member 3 | *** | | * | -2,7 |
| *SULT1E1* | | sulfotransferase family 1E, estrogen-preferring, member 1 | *** | | * | -2,6 |
| *SULT2B1* | | sulfotransferase family, cytosolic, 2B, member 1 | *** | | -1,7 | -2,3 |
| *TNF* | | tumor necrosis factor | *** | | * | 3,5 |
| *UGT1A6* | | UDP glucuronosyltransferase 1 family, polypeptide A6 | *** | | * | -1,6 |
| *UGT1A9 (includes others)* | | UDP glucuronosyltransferase 1 family, polypeptide A8 | *** | | * | -1,5 |
| *UGT2B7* | | UDP glucuronosyltransferase 2 family, polypeptide B7 | *** | | * | -2,4 |
| *UGT2B10* | | UDP glucuronosyltransferase 2 family, polypeptide B10 | *** | | * | -2,3 |
| *UGT2B11* | | UDP glucuronosyltransferase 2 family, polypeptide B11 | *** | | * | -1,9 |
| *UGT2B15* | | UDP glucuronosyltransferase 2 family, polypeptide B15 | *** | | -2,0 | -3,3 |
| *UST* | | uronyl-2-sulfotransferase | *** | | * | 1,9 |
|  | |  |  | |  |  |
|  | |  |  | |  |  |
|  | | **Proteomic response** |  | |  |  |
| ***Symbol*** | | **Name** | **Swiss Prot number** | | **Fold Change (3,0 µg/cm²)** | **Fold Change (6,0 µg/cm²))** |
|  | |  |  | |  |  |
| ***Xenobiotic Metabolism Signaling*** | | |  | |  |  |
| AKR1C3 | | aldo-keto reductase family 1, member C3 | P42330 | | 19,0 | 22,0 |
| ALDH1A1 | | aldehyde dehydrogenase 1 family, member A1 | P00352 | | 20,4 | 21,5 |
| GSTP1 | | glutathione S-transferase pi 1 | P09211 | | 11,0 | 11,3 |
| HSP90AA1 | | heat shock protein 90kDa alpha (cytosolic), class A member 1 | P07900 | | 38,3 | 32,0 |
| HSP90AB1 | | heat shock protein 90kDa alpha (cytosolic), class B member 1 | P08238 | | 14,2 | 19,4 |
|  | |  |  | |  |  |
|  | |  |  | |  |  |
| ***Oxidative Stress Response*** | | | | | | |
| ACTB | | actin, beta | P60709 | | 7,1 | 7,9 |
| GSR | | glutathione reductase | P00390 | |  | 4,3 |
| GSTP1 | | glutathione S-transferase pi 1 | P09211 | | 11,0 | 11,3 |
| HSP90AA1 | | heat shock protein 90kDa alpha (cytosolic), class A member 1 | P07900 | | 38,3 | 32,0 |
| HSP90AB1 | | heat shock protein 90kDa alpha (cytosolic), class B member 1 | P08238 | | 14,2 | 19,4 |
| LDHA | | lactate dehydrogenase A | P00338 | | 32,7 | 34,3 |
| LDHB | | lactate dehydrogenase B | P07195 | | 31,0 | 31,7 |
| PRDX1 | | peroxiredoxin 1 | Q06830 | | 6,4 | 6,8 |
| PRDX6 | | peroxiredoxin 6 | P30041 | | 2,3 | 3,3 |
| TXNRD1 | | thioredoxin reductase 1 | Q16881 | | 13,0 | 12,7 |
| VCP | | valosin containing protein | P55072 | | 19,3 | 16,0 |
|  | |  |  | |  |  |
| ***Pulmonary inflammation*** | | |  | |  |  |
| ACTB | | actin, beta | P60709 | | 7,1 | 7,9 |
| ALDOA | | aldolase A, fructose-bisphosphate | P04075 | | 22,3 | 20,0 |
| ANXA1 | | annexin A1 | P04083 | | 14,8 | 14,8 |
| ENO1 | | enolase 1, (alpha) | P06733 | | 25,3 | 25,5 |
| GAPDH | | glyceraldehyde-3-phosphate dehydrogenase | P04406 | | 24,7 | 28,7 |
| HSPA5 | | heat shock 70kDa protein 5 (glucose-regulated protein, 78kDa) | P11021 | | 8,0 | 7,7 |
| PRDX1 | | peroxiredoxin 1 | Q06830 | | 6,4 | 6,8 |
| PRDX6 | | peroxiredoxin 6 | P30041 | | 2,3 | 3,3 |
| TKT | | transketolase | P29401 | | 14,7 | 17,4 |
| TPI1 | | triosephosphate isomerase 1 | P60174 | | 13,8 | 16,3 |
| TUBB | | tubulin, beta class I | P07437 | | 32,0 | 36,3 |
| THBS1 | | thrombospondin 1 | P07996 | | -4,5 | -9,0 |
