# Supplementary material for: High-throughput, quantitative assessment of the effects of low-dose silica nanoparticles on lung cells: grasping complex toxicity with a great depth of field
Source: BMC Genomics. 2015 Apr 18;16(1):315. doi: 10.1186/s12864-015-1521-5 (PMC4404697; doi:10.1186/s12864-015-1521-5)
Supplement: Additional file 4: Table S4. — List of primers used in qRT-PCR. Differential analysis of transcripts from cells exposed to 6.0 μg/cm2 SiO2 NPs for 72 h versus unexposed cells was performed by qRT-PCR with the Sybr Green PCR Master Mix (Finzyme) kit according to the manufacturer's instructions on Opticon II (Biorad). This pool of genes represents the “coagulation system” pathway. [file 12864_2015_1521_MOESM4_ESM.docx]

**Table S4. List of primers used for qRT-PCR**

| **Gene ID** | **Forward primer** | **Reverse primer** | **Amplicon (bp)** |
| --- | --- | --- | --- |
| *FGA* | 5’ TGCATCACAGATGAACCCCA 3’ | 5’ CAAGGCAATACGTGTGAGTGT 3’ | 204 |
| *F2* | 5’ GCATCGTCTCATGGGGTGAA 3’ | 5’ CCAGAATATGAGTGGCCCCC 3’ | 132 |
| *PLAU* | 5’ ACTCCAAAGGCAGCAATGAAC 3’ | 5’ GACAGTGGCAGAGTTCCAGG 3’ | 248 |
| *FGG* | 5' TCACGCTGGCCATCTCAATG 3’ | 5’ TCTGGTCTGACCTGTTTGGC 3’ | 216 |
| *PROC* | 5’ TCTTCGTCCACCCCAACTAC 3’ | 5’ GGTTTCTCTTGGCCTCCTTC 3’ | 213 |
| *F7* | 5’AACTGTCCTGGCACCAAATC 3’ | 5’TGCCTCTGCCTCTCATCTTT 3’ | 234 |
